# The Use of Cannabidiol in Cutaneous Wound Healing: A Systematic Review

**DOI:** 10.1096/fj.202601469R

**Published:** 2026-10-13

**Authors:** João Henrique Ferreira França, Renale Beserra de Melo Freitas, Geovanne Lucas Rodrigues Rosendo, Mariana Pereira Carvalho, Glicia Maria de Oliveira, Júlio César Claudino dos Santos, Fernanda das Chagas Angelo Mendes Tenorio, Jaiurte Gomes Martins da Silva

**Affiliations:** ^1^ Universidade Federal de Pernambuco (UFPE) Recife PE Brazil; ^2^ Universidade Federal da Paraíba (UFPB) João Pessoa PB Brazil; ^3^ Universidade Federal de Alagoas (UFAL) Arapiraca AL Brazil; ^4^ Centro Acadêmico de Vitória (CAV) Universidade Federal de Pernambuco (UFPE) Vitória de Santo Antão PE Brazil; ^5^ Universidade Federal Fluminense (UFF) Niterói RJ Brazil

**Keywords:** cannabidiol, regeneration, skin, systematic review, wound healing

## Abstract

Cutaneous wounds represent a significant health problem due to their high prevalence, functional impact, and effects on quality of life. Cannabidiol (CBD), a non‐psychoactive compound derived from *Cannabis sativa* , has been investigated for its anti‐inflammatory, antioxidant, and regenerative properties, suggesting potential therapeutic applications in wound healing. To systematically evaluate the available evidence on the efficacy of cannabidiol in cutaneous wound healing. This is a qualitative systematic review conducted in accordance with PRISMA guidelines and registered in PROSPERO (CRD420261333317). The search was performed in PubMed, EMBASE, Web of Science, Scopus, Cochrane Library, LILACS, and MEDLINE, without restriction on publication date. In vivo studies and clinical trials investigating the use of CBD were included. Study selection was carried out by independent reviewers, and the risk of bias was assessed using the RoB 2 and SYRCLE tools. A total of 190 studies were identified, of which six were included. Four of the five included studies reported beneficial effects of CBD, including increased wound closure, enhanced collagen deposition, stimulation of angiogenesis, and reduced inflammation, as well as improvements in histological and biochemical parameters associated with tissue repair. The studies also indicated increased fibroblast migration and reduced oxidative stress. However, the results were heterogeneous, and one study found no significant differences compared to the control group. Cannabidiol shows promising therapeutic potential in wound healing; however, the current evidence remains limited, and more robust clinical studies are required.

## Graphical abstract

Proposed mechanisms by which cannabidiol (CBD) promotes cutaneous wound healing. CBD modulates inflammatory and oxidative pathways, enhances fibroblast migration and proliferation, stimulates collagen deposition and extracellular matrix remodeling, and promotes angiogenesis, collectively contributing to improved tissue repair.

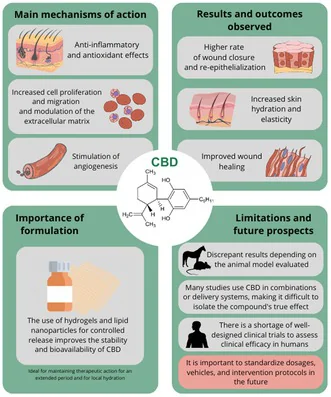

## 1 Introduction

Skin wounds are defined as a loss of continuity in the epithelial tissue that compromises the integrity of the skin, primarily the epidermis, and may also affect deeper layers, such as the dermis and subcutaneous tissue [1]. Their etiology is multifactorial and may be related to mechanical trauma, burns, infections, vascular disorders, and chronic diseases. In addition, clinical interventions can result in the formation of these wounds, especially when they involve incisions or invasive procedures [2].

These lesions trigger biological processes aimed at tissue repair. However, their effects are not limited to structural changes; they also have significant functional implications, such as pain, limited mobility, and changes in sensation [3]. In addition to physical impacts, skin conditions can affect individuals' psychological well‐being, as characteristic symptoms, combined with aesthetic factors, can directly influence self‐image, self‐esteem, and even social relationships [4]. Thus, they can have a significant impact on quality of life.

The wound healing process occurs through well‐established phases: inflammation, proliferation, and tissue remodeling. However, alterations in any of these stages can lead to complications such as delayed healing, infections, or the formation of pathological scars, prolonging treatment and compromising the individual's functional recovery [5]. From an epidemiological perspective, chronic wounds represent a substantial public health challenge. Recent estimates indicate that chronic wounds affect approximately 10.5 million U.S. Medicare beneficiaries, corresponding to nearly 2.5% of the U.S. population and continue to impose a considerable clinical and economic burden [6, 7].

In this context, various therapeutic approaches can be used to promote the healing process, including local care, the use of appropriate dressings, and the management of factors that may interfere with tissue repair [8]. In addition, strategies aimed at accelerating this process have been investigated, such as the use of biomaterials and substances with anti‐inflammatory and regenerative properties, seeking to optimize the wound environment and promote more efficient recovery [6, 9]. In light of this, the present study aims to analyze the feasibility, efficacy, and potential advantages of using cannabidiol in the treatment of skin wounds, compared to conventional therapies.

## 2 Materials and Methods

This is a qualitative systematic literature review conducted in accordance with the PRISMA (Preferred Reporting Items for Systematic Reviews and Meta‐Analyses) guidelines. The study was previously registered on the PROSPERO platform under number CRD420261333317.

This review was designed to identify, compile, and critically analyze the available scientific evidence on the efficacy of cannabidiol in the healing of skin wounds, both in in vivo (animal) models and in human clinical trials, recognizing the translational importance of these studies in the field of bioactive compounds and regenerative medicine.

### 2.1 Study Selection

The publications were identified through a systematic search of relevant electronic databases in the biomedical and health fields, including PubMed (U.S. National Library of Medicine), EMBASE, Web of Science, Scopus, the Cochrane Library, LILACS, and MEDLINE. The search included all studies published up to March 31, 2026, and the final database search was performed on that date.

The search strategy was designed to be comprehensive, with the aim of maximizing sensitivity and minimizing the risk of excluding potentially relevant studies. Thus, no restrictions were applied regarding publication date or study type at this stage. The search terms used included “cannabidiol,” “cannabinoids,” “cannabinol,” and “cannabis,” as well as “skin wound healing,” “skin wound closure techniques,” “skin regeneration,” and “skin wound,” combined using the Boolean operators AND and OR. The search strategies were adapted for each database, considering the title, abstract, and subject fields.

### 2.2 Eligibility Criteria

The inclusion criteria encompassed in vivo experimental studies and clinical trials investigating the therapeutic use of cannabidiol in skin wounds, available in full in the selected databases, published in English, Portuguese, and/or Spanish, with no restriction on publication date. Studies presenting quantitative or qualitative data related to wound healing were considered eligible, including outcomes such as inflammatory response, tissue regeneration, and structural properties of dressings, regardless of the animal species used. Additionally, studies that used cannabidiol alone as an intervention or as a comparative/control group were included.

The exclusion criteria, in turn, were as follows: (1) studies conducted exclusively in vitro, as they do not adequately represent the complexity of the wound healing process in living organisms; (2) studies that did not present relevant quantitative or qualitative data on the wound healing process; (3) studies that did not use cannabidiol in isolation as an intervention or in a comparison group, making it impossible to specifically evaluate its effects; (4) literature reviews, theses, dissertations, book chapters, and conference proceedings, since this review focuses on the analysis of primary evidence derived from original research.

### 2.3 Selection Process

The screening and selection of studies followed the PRISMA protocol. After conducting the search strategies, all references were imported into the Rayyan online platform [10], developed to optimize literature reviews. This tool facilitated the independent evaluation of articles by three reviewers, with the aim of ensuring the rigor of the analysis. Although the software has an automated duplicate resolution system, this evaluation was performed manually by the authors as an essential preliminary step to screening. Subsequently, eligibility criteria were applied using a blinding system available on Rayyan. Articles that met these criteria were selected for full‐text reading, as the analysis up to that point had been limited to the title, abstract, and publication information. Additionally, a flowchart of the study selection process was created to facilitate understanding of the stages of identification, screening, eligibility, and inclusion of articles.

### 2.4 Data Collection and Extraction

Data from the included studies were independently extracted by the same three reviewers who performed the study selection. Any discrepancies were discussed and resolved by consensus. The variables extracted included: authors, year of publication, country, journal, study type (preclinical or clinical), sample, type of cannabidiol used, study period, interventions, outcomes assessed, main results, and identified limitations. This information was organized into Tables 1, 2, 3 to facilitate comparative analysis and qualitative synthesis.

**TABLE 1:** Study identification.

Author and year of publication	Article title	Journal	Type of study	Study location
Palmieri et al. 2019 [11]	A therapeutic effect of cbd‐enriched ointment in inflammatory skin diseases and cutaneous scars.	Clinical Trial.	A retrospective, anecdotal, and spontaneous study.	Second Opinion Medical Network—Modena, Italy.
Akarsu and Akarsu 2024 [12]	Therapeutic potential of cannabis for surgical wound healing in rats.	Veterinarni Medicina.	Experimental study using an animal model (male albino Wistar rats).	Center for Experimental Research and Medical Biochemistry Laboratories at Erciyes University, Kayseri, Turkey.
McIver et al. 2020 [13]	Effects of topical treatment of cannabidiol extract in a unique manuka factor 5 manuka honey carrier on second intention wound healing on equine distal limb wounds: a preliminary study.	Australian Veterinary Journal.	Experimental Project	University of Sydney Veterinary Hospital, Camden, Australia, in collaboration with the University of Queensland, Gatton, Australia.
Lapmanee et al. 2024 [14]	Cannabidiol‐Loaded Lipid Nanoparticles Incorporated in Polyvinyl Alcohol and Sodium Alginate Hydrogel Scaffold for Enhancing Cell Migration and Accelerating Wound Healing.	Gels (MDPI).	Preclinical experimental study involving in vitro assays (fibroblast cells) and in vivo assays (Wistar rats).	Pathumthani, Bangkok, Phitsanulok, and Nonthaburi, Thailand.
Zheng et al. 2022 [15]	A cannabidiol‐containing alginate based hydrogel as novel multifunctional wound dressing for promoting wound healing	Biomaterials Advances	Preclinical experimental study using an animal model (rats) and in vitro experiments to characterize the biomaterial.	The study was conducted at Southern University of Science and Technology in Shenzhen, China, in collaboration with other Chinese and Canadian institutions.

**TABLE 2:** Characteristics of the studies.

Author and year of publication	Objective	Sample	Study period
Palmieri et al. 2019 [11]	To investigate the therapeutic effect of CBD ointment in the treatment of severe chronic skin diseases and/or on their prognosis.	Patients with psoriasis (*n* = 5), atopic dermatitis (*n* = 5), and skin scars (*n* = 10); total *n* = 20 (14 women, 6 men).	Each participant used the CBD‐enriched ointment for 3 months, applying it twice a day to the affected areas of the skin.
Akarsu and Akarsu 2024 [12]	To evaluate the efficacy of hemp essential oils, hemp seeds, and CBD compared to 10% povidone‐iodine, which is routinely used in abdominal suturing during surgery in rats.	Male Wistar rats (*n* = 48), a cutaneous incision model, divided into 4 experimental groups.	The experimental period lasted 21 days, with wound care provided for 10 days and histological evaluations performed on days 1, 2, 3, 10, and 21 post‐surgery.
McIver et al. 2020 [13]	To investigate the potential of CBD extract to modulate secondary healing of distal limb wounds in horses.	Adult Standardbred horses (*n* = 6), with a skin lesion on the forelimbs.	The study's trial period lasted 42 days (6 weeks), during which the wounds were monitored and photographed periodically to assess healing.
Lapmanee et al. 2024 [14]	To evaluate the formulation of CBD/LNPs incorporated into PVS/SA hydrogels and their application in promoting wound healing.	Human fibroblasts (in vitro) and adult male Wistar rats (*n* = 15), a skin wound model.	In vivo evaluation: Days 3, 7, 14, 21, and 28. Nanoparticle stability: up to 2 months. Cell‐based assays: up to 48 h.
Zheng et al. 2022 [15]	To develop and evaluate an alginate‐based hydrogel containing cannabidiol (CBD) and zinc ions (Zn^2+^) as a multifunctional wound dressing to promote the healing of skin wounds, leveraging its antioxidant, anti‐inflammatory, antibacterial, and pro‐angiogenic properties.	Male Sprague–Dawley rats (*n* = 24), an acute skin wound model, divided into 4 experimental groups.	On Days 0, 3, 7, 10, and 14 after surgery, the wound healing status was recorded and photographed, and skin tissue was collected from the wound site after the rats were euthanized on Days 7 and 14.

**TABLE 3:** Key findings of the studies.

Author and year of publication	Interventions	Outcomes assessed	Main results	Limitations
Palmieri et al. 2019 [11]	Topical application of a CBD‐containing ointment (CBD concentration not reported), applied twice daily (morning and evening) for 3 months, without concurrent use of other topical products.	Skin hydration, transepidermal water loss (TEWL), skin elasticity, photographic assessment, and clinical scores (SCORAD, ADI, PASI).	After 3 months, a significant increase in skin hydration (~5%–7%), a reduction in TEWL, and an improvement in elasticity (~17%–27%) were observed in all areas evaluated (*p* < 0.01–0.001).	The main limitations of the study include its uncontrolled, retrospective design, as well as the small, clinically heterogeneous cohort.
Akarsu and Akarsu 2024 [12]	Abdominal incision model in rats (4 cm), with sutures. Daily topical application of the specific treatment for each group to the wound site and adjacent area for 10 days, with sterile dressings and standardized postoperative care.	Histological assessment of wound healing (Days 1, 2, 3, 10, and 21) and analysis of biomarkers (EGF, MCP‐1, bFGF, IL‐8, COL‐1) related to inflammation and tissue repair.	The treated groups showed faster and more complete healing (by Day 10), with better tissue organization and no visible scarring. Less inflammation was observed compared to the control group. There were no significant differences in EGF, bFGF, IL‐8, and COL‐1; MCP‐1 showed a reduction in a specific group.	Not described.
McIver et al. 2020 [13]	Daily topical application of 1% CBD in Manuka honey (UMF 5), isolated Manuka honey (UMF 5), UMF 20 honey, or saline solution to a skin wound model for up to 42 days, using standardized dressings.	Wound area over time (image analysis), healing rate (cm^2^/day), and total time to complete closure.	There was no difference between the groups in terms of wound area, healing rate, or total time to closure. A significant reduction in wound area was observed over time in all groups (days 14–42 vs. initial days; *p* < 0.05), with a slowing of the healing process after Day 21.	Preliminary study; lack of detailed discussion of limitations; need for validation in further studies.
Lapmanee et al. 2024 [14]	Each rats had two wounds: one treated with a PVA/alginate hydrogel containing CBD encapsulated in lipid nanoparticles (CBD/LNP‐PVA/SA) and the other with a hydrogel (PVA/SA) without CBD, serving as an internal control.	Physicochemical characterization of the hydrogel (swelling, release, and nanoparticle properties), in vitro evaluation (cytotoxicity, oxidative stress, cell migration), and in vivo evaluation (wound healing, contraction, histological parameters: collagen, dermal thickness, angiogenesis, and inflammation).	Lipid nanoparticles containing CBD exhibited greater stability and lower cytotoxicity, as well as increased fibroblast migration and reduced oxidative stress. In vivo, wound healing was accelerated (~35% by Day 28), accompanied by increased collagen deposition, dermal thickness, and vascularization, along with reduced inflammatory infiltration.	Exclusive use of male animals; lack of assessment of inflammatory cytokines; failure to quantify CBD release; need for translational validation.
Zheng et al. 2022 [15]	The study involved the development and application of a multifunctional hydrogel for wound dressings containing cannabidiol (CBD) incorporated into an alginate matrix and zinc ions (Zn^2+^).	Wound closure and reduction in wound area over time, in conjunction with histological analysis (granulation tissue, epithelialization, collagen, angiogenesis, and inflammation), as well as assessment of antibacterial and antioxidant activity.	Treatment with CBD/Alg@Zn promoted faster wound closure and a smaller residual wound area (Day 10). An increase in granulation tissue formation, epithelialization, and tissue organization was observed, along with increased collagen deposition and angiogenesis, accompanied by a reduction in inflammation. It also exhibited antibacterial and antioxidant activity.	Not described.

### 2.5 Assessment of Risk of Bias

The methodological quality and risk of bias of the included studies were assessed according to each study's design. Non‐randomized studies were assessed using the Risk Of Bias In Non‐randomized Studies—of Interventions, Version 2 (ROBINS‐I V2) tool, while in vivo preclinical studies were assessed using the SYRCLE (Systematic Review Centre for Laboratory Animal Experimentation) tool.

The ROBINS‐I V2 tool assesses six domains of bias in non‐randomized studies of interventions: bias due to confounding, bias in the classification of interventions, bias in the selection of participants into the study (or into the analysis), bias due to missing data, bias arising from measurement of the outcome, and bias in the selection of the reported result. Each domain is judged as presenting low, moderate, serious, or critical risk of bias, leading to an overall risk‐of‐bias assessment for each study [16].

The SYRCLE tool, on the other hand, assesses six domains (selection, performance, detection, attrition, reporting, and other biases), allowing studies to be classified as having low, high, or uncertain risk of bias [17]. The analysis was conducted independently by three reviewers, with discrepancies resolved by consensus. The overall risk of bias in the experimental studies was determined based on the synthesis of the assessed domains, considering the prevalence and relevance of the identified biases.

### 2.6 Data Synthesis

Because of methodological and outcome heterogeneity among the included studies, a quantitative meta‐analysis was not feasible. Therefore, the results were synthesized descriptively and qualitatively, highlighting the effects of cannabidiol (CBD) on wound healing, including inflammation modulation, wound closure, and tissue regeneration.

## 3 Results

### 3.1 Study Selection

A search of the selected databases, using the defined health‐related keywords, yielded a total of 190 records. After removing duplicates (*n* = 61), the remaining studies underwent screening through the review of titles and abstracts, conducted independently by pairs of reviewers, resulting in 19 articles selected for full‐text reading. After evaluating the full texts, 14 studies were excluded for failing to meet the eligibility criteria. In the end, 5 studies were included in this review. The study selection process is presented in the PRISMA flowchart (Figure 1). Among the included studies, 1 was a randomized clinical trial (33.3%) and 4 were in vivo preclinical studies (66.7%); furthermore, the years of publication range from 2019 to 2025.

**FIGURE 1:**
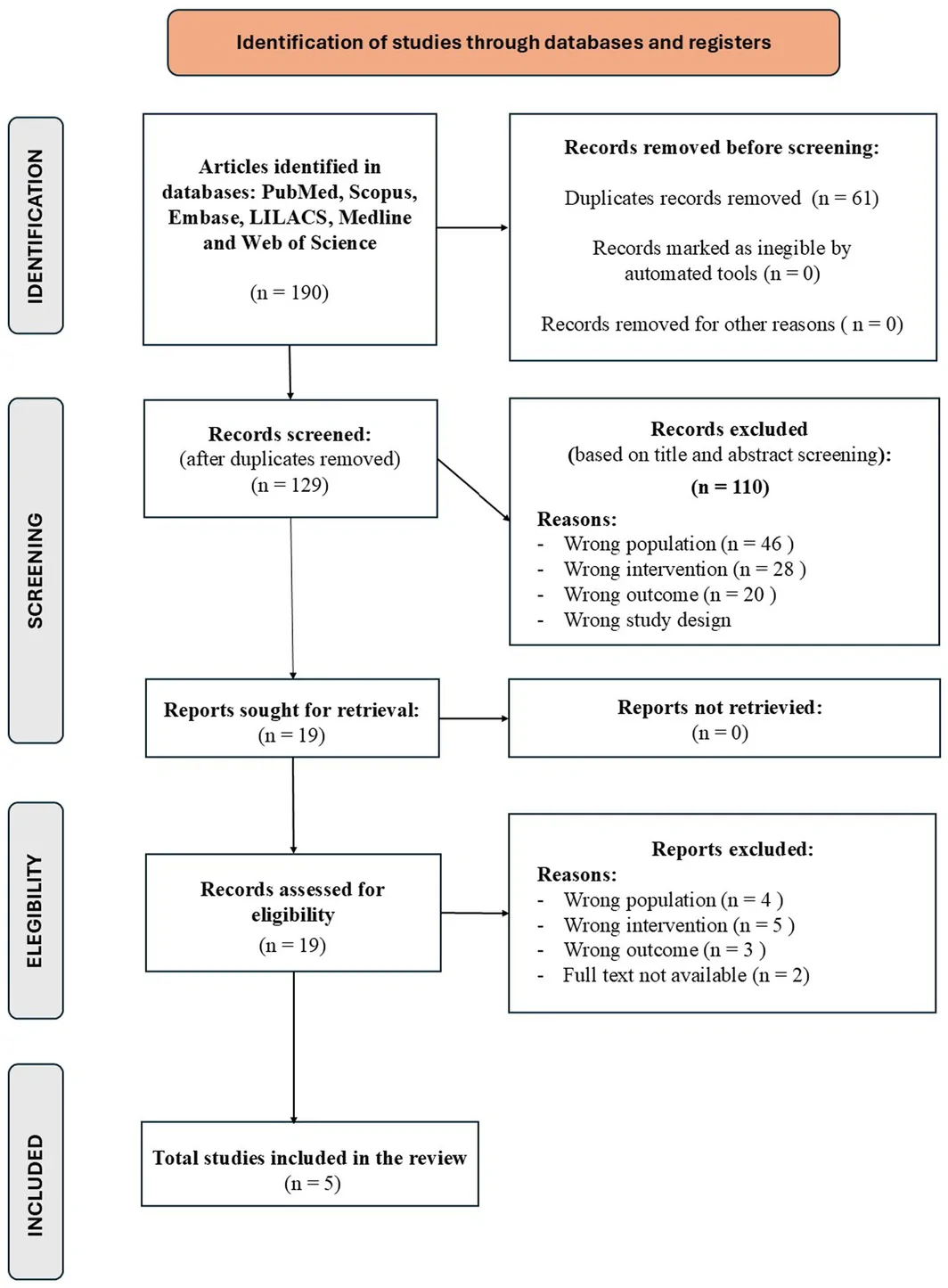
PRISMA flow diagram of study selection.

### 3.2 Characteristics of Included Studies (Tables 1, 2, 3)

#### 3.2.1 Risk of Bias Assessment

The assessment of the risk of bias in the included studies revealed heterogeneity in methodological quality, with a predominance of moderate risk among the analyzed studies. Overall, the main domains responsible for methodological limitations were related to the randomization process, blinding, and reporting of outcomes.

In the study by Palmieri et al. [11], the assessment of methodological quality using the ROBINS‐I V2 tool classified the study as having a critical risk of bias. Although the domains related to bias in intervention classification (D2), bias in participant selection (D3), and bias due to missing data (D4) were classified as having a low risk of bias, the overall classification was a critical risk. This final judgment resulted primarily from the limitations identified in the domains related to bias due to confounding factors (D1‐A and D1‐B), bias in outcome measurement (D5), and bias in the selection of reported outcomes (D6).

In the preclinical studies evaluated using the SYRCLE tool (Table 4), the main critical issues involved the absence or insufficient description of allocation concealment (D2) and blinding of outcome assessors (D3), aspects that may introduce selection and detection biases. Additionally, in some studies, a risk related to incomplete outcome data (D4/D5) was observed, suggesting possible losses or exclusions that were not adequately justified. The study by Akarsu and Akarsu [12] stood out for presenting low risk in most domains, indicating greater methodological rigor. In contrast, the studies by McIver et al. [13], Lapmanee et al. [14], and Zheng et al. [15] presented recurring limitations in these domains, which justified their classification as moderate risk.

**TABLE 4:** Risk of bias assessment of preclinical studies using the SYRCLE tool.

SYRCLE							
Author/Year	D1	D2	D3	D4	D5	D6	Overall risk
Akarsu and Akarsu 2024 [12]	?	?	+	+	+	+	Low
McIver et al. 2020 [13]	+	−	?	+	+	?	Moderate
Lapmanee et al. 2024 [14]	+	?	−	+	+	?	Moderate
Zheng et al. 2022 [15]	+	?	−	+	+	+	Moderate

*Note:* Risk of bias assessment: (+) low risk of bias, indicated in green; (−) high risk of bias, indicated in red; and (?) unclear risk of bias, indicated in gray.

### 3.3 Effects of Cannabidiol on Wound Healing

Overall, the included studies investigated the effects of cannabidiol (CBD) on different wound‐healing models, including human clinical trials and preclinical in vivo and in vitro experimental studies. The interventions primarily involved topical formulations containing CBD alone or combined with biomaterials, such as hydrogels and lipid nanoparticles. The main outcomes assessed included wound closure rate, biophysical skin parameters, inflammatory response, cell proliferation, angiogenesis, and tissue remodeling, as detailed in Table 3.

Regarding macroscopic outcomes and biophysical parameters of the skin, Palmieri et al. [12] observed a significant increase in skin hydration after 3 months of treatment with topical CBD, with variations ranging from approximately 5.5%–6.9% in the evaluated areas. There was also a reduction in transepidermal water loss (TEWL), with improvements ranging from 17.6% to 28.1%, as well as an increase in skin elasticity ranging from 17.1% to 27.1%.

In in vivo experimental models, Lapmanee et al. [14] demonstrated an increase in the wound closure rate over time in groups treated with a hydrogel containing CBD encapsulated in lipid nanoparticles, with an approximate 35% improvement in healing by Day 28. These findings were accompanied by increased collagen deposition, greater dermal thickness, and a higher number of blood vessels, as well as reduced infiltration of inflammatory cells. Similarly, Zheng et al. [15] reported a more pronounced reduction in wound area in the groups treated with hydrogel containing CBD combined with alginate and zinc, with differences observed starting on Day 10, as well as increased granulation tissue formation, faster epithelialization, increased collagen deposition, and higher vascular density.

Regarding cellular and biochemical outcomes, Lapmanee et al. [14] observed, in in vitro assays, increased fibroblast migration and reduced levels of intracellular reactive oxygen species in cells treated with formulations containing CBD. Zheng et al. [15] described reduced infiltration of inflammatory cells in the treated groups, as assessed by histological evaluation of the tissue. On the other hand, Akarsu and Akarsu [12] did not observe significant differences between the groups regarding plasma levels of EGF, BFGF, IL‐8, and COL‐1 (*p* > 0.05), although differences were identified in MCP‐1 levels, with reduced values in the CBD‐treated group compared to some experimental groups. In the histological evaluation, differences between the groups were observed over time, with variations in the progression of the healing process among the treatments.

Contradictory results were observed in certain experimental models. McIver et al. [13], in an equine model of secondary‐intent healing, found no significant differences between the groups treated with CBD and those treated with manuka monofactor or saline control regarding wound area on the first day after wound creation, nor on any of the treatment days, although between Days 14 and 42 the mean area was smaller than the means for Days 1 and 7 in all intervention groups. Furthermore, they observed no differences in the average healing rate (cm^2^/day) in any of the groups, nor in the analysis of the total time to wound closure. As a result, over the 42 days of treatment, there was a reduction in the average wound area, as well as a healing process, but no significant difference was observed among the treatment modalities used.

Overall, the included studies presented heterogeneous results regarding the effects of cannabidiol on the wound healing process. In summary, effects related to the evaluated outcomes were observed in different experimental models, although not all studies demonstrated significant differences compared to the control groups.

## 4 Discussion

To the best of our knowledge, this is the first systematic review to compile evidence on the use of cannabidiol (CBD) as a therapeutic strategy in the treatment of skin wounds. Although there are studies addressing medical cannabis and other cannabinoids, the present study focuses exclusively on CBD, gathering information on the experimental and methodological approaches employed to optimize the healing process. Overall, the findings indicate that the application of this bioactive compound is associated with positive effects on healing in most of the included studies, although neutral results have also been reported. In this regard, the data suggest that CBD has therapeutic potential in wound healing, but with inconsistencies among the studies, particularly due to the heterogeneity of the experimental models, sample types, and intervention protocols used.

The main differences among the included studies lie primarily in the types of samples used, which comprised humans, different rat strains (Wistar and Sprague–Dawley), and Standardbred horses. Additional heterogeneity was observed in the wound models investigated (psoriasis, atopic dermatitis, scars, and acute incisions), treatment duration, and CBD delivery vehicles, including ointments, hydrogels, lipid nanoparticles, and Manuka honey. This methodological heterogeneity highlights the need for caution when interpreting the findings of this review, as it hinders direct comparisons across studies and limits the ability to draw more robust conclusions regarding the efficacy of CBD in wound healing.

The beneficial effects of CBD observed in the included studies may be partially explained by its interaction with multiple receptors and signaling pathways associated with the endocannabinoid system. CBD modulates CB1 and indirectly enhances CB2 receptor activation, contributing to pain relief, attenuation of inflammation, and reduced production of pro‐inflammatory cytokines, including TNF‐α, IL‐1β, and IL‐6 [18]. In addition, CBD activates and subsequently desensitizes TRPV1, regulating keratinocyte migration and re‐epithelialization, while acting as a PPARγ agonist to inhibit the NF‐κB signaling pathway, thereby reducing oxidative stress and promoting fibroblast proliferation and connective tissue remodeling. Collectively, these mechanisms are consistent with the enhanced collagen deposition and accelerated wound closure observed in the included studies [18].

The available evidence suggests that CBD may reduce inflammatory infiltration and, consequently, the release of reactive oxygen species (ROS), which may be associated with the anti‐inflammatory effect of cannabidiol. In this context, Atalay et al. [19] show that CBD plays a significant role in modulating the inflammatory response and oxidative stress. This regulation is directly related to the healing process, since exacerbated inflammation can intensify oxidative stress and prolong healing time.

In addition to these anti‐inflammatory effects, CBD was associated with increased fibroblast migration and collagen deposition. These events promote accelerated tissue regeneration, contributing particularly to the proliferative and remodeling phases, in addition to increasing the rate of wound closure over time. In agreement, Gerasymchuk et al. [20] state that cannabidiol influences fibroblast recruitment, promoting wound closure and the organization of the extracellular matrix. Furthermore, it has been observed that CBD can modulate growth factors, such as VEGF, which are directly related to angiogenesis and collagen deposition, as noted by Klinsang et al. [21].

Although promising results have been observed regarding the use of cannabidiol, considerable variability has been identified in formulations and application methods, which may influence the healing process in different ways. Nevertheless, Millar et al. [22] highlight that controlled‐release strategies, such as those employed in the included studies, increase the compound's bioavailability and stability, contributing to the maintenance of its therapeutic effects even across different routes of administration. Additionally, the most significant results were observed in hydrogel‐based formulations, which appear to demonstrate superior performance, possibly by promoting sustained release of the compound, maintaining local moisture, and facilitating better interaction with the injured tissue, as described by Boateng and Catanzano [23].

Although the available evidence suggests promising effects of cannabidiol (CBD) on wound healing, it does not support its replacement of established standard therapies. Conventional approaches, including negative pressure wound therapy (NPWT), hydrocolloid dressings, recombinant growth factors, hydrogels, and skin grafts, remain the standard of care because of their proven clinical efficacy [24]. In contrast, evidence for CBD is still predominantly based on in vitro and preclinical studies, with limited clinical trials directly comparing its efficacy with standard treatments. Therefore, CBD may be better considered an adjunctive therapy that modulates the wound microenvironment by reducing inflammation and promoting extracellular matrix remodeling, thereby complementing conventional wound management [25].

Although this review focused exclusively on CBD, other phytocannabinoids have also demonstrated potential in skin repair. Emerging evidence suggests that cannabigerol (CBG) exhibits anti‐inflammatory and antimicrobial properties, whereas tetrahydrocannabinol (THC) may contribute to tissue repair through immunomodulatory and analgesic effects. Cannabinol (CBN) has been less extensively investigated but may also exert anti‐inflammatory activity. Nevertheless, current evidence for these phytocannabinoids remains limited, and comparative studies are needed to determine their relative efficacy, mechanisms of action, and potential advantages over CBD in wound healing applications [2, 24, 26].

Despite the promising findings, several translational barriers still limit the clinical application of CBD for wound healing. According to Shah et al. [26], the low and variable bioavailability of CBD, which depends on the route of administration and formulation, together with the lack of standardized dose–response protocols, remains a major challenge for achieving consistent therapeutic outcomes. Although nanoformulations and hydrogel‐based delivery systems have shown potential to improve local drug delivery and tissue penetration, robust clinical evidence is still limited. Furthermore, long‐term safety data and standardized regulatory frameworks are required before CBD‐based therapies can be routinely incorporated into clinical practice [26].

The available evidence suggests that the effects of CBD on wound healing may vary according to the experimental model, formulation, dosage, and route of administration, highlighting important limitations in the translation and extrapolation of current findings. Experimental studies included in this review reported improvements in inflammatory parameters and wound closure in humans and murine models. These findings are consistent with the biological principles of wound healing described by Guo and DiPietro [27]. However, investigations in larger animal models, such as horses, have not consistently reproduced these beneficial effects, suggesting that physiological and metabolic differences, as well as variations in the tissue microenvironment, may influence treatment outcomes across species [28]. Thus, divergent results suggest that factors such as dose, vehicle, experimental model, and follow‐up time impact the outcomes. These inconsistencies highlight the need for greater methodological standardization and comparative studies.

Overall, the available evidence supports the potential of CBD as an adjunctive strategy for wound healing due to its anti‐inflammatory, antioxidant, and regenerative properties. To facilitate the interpretation of the findings, Figure 2 provides a schematic overview of the proposed mechanisms by which cannabidiol may promote cutaneous wound healing. Based on the evidence synthesized in this review, CBD appears to modulate key molecular pathways involved in inflammation, oxidative stress, cell migration, angiogenesis, collagen deposition, and extracellular matrix remodeling, ultimately creating a microenvironment favorable to tissue repair.

**FIGURE 2:**
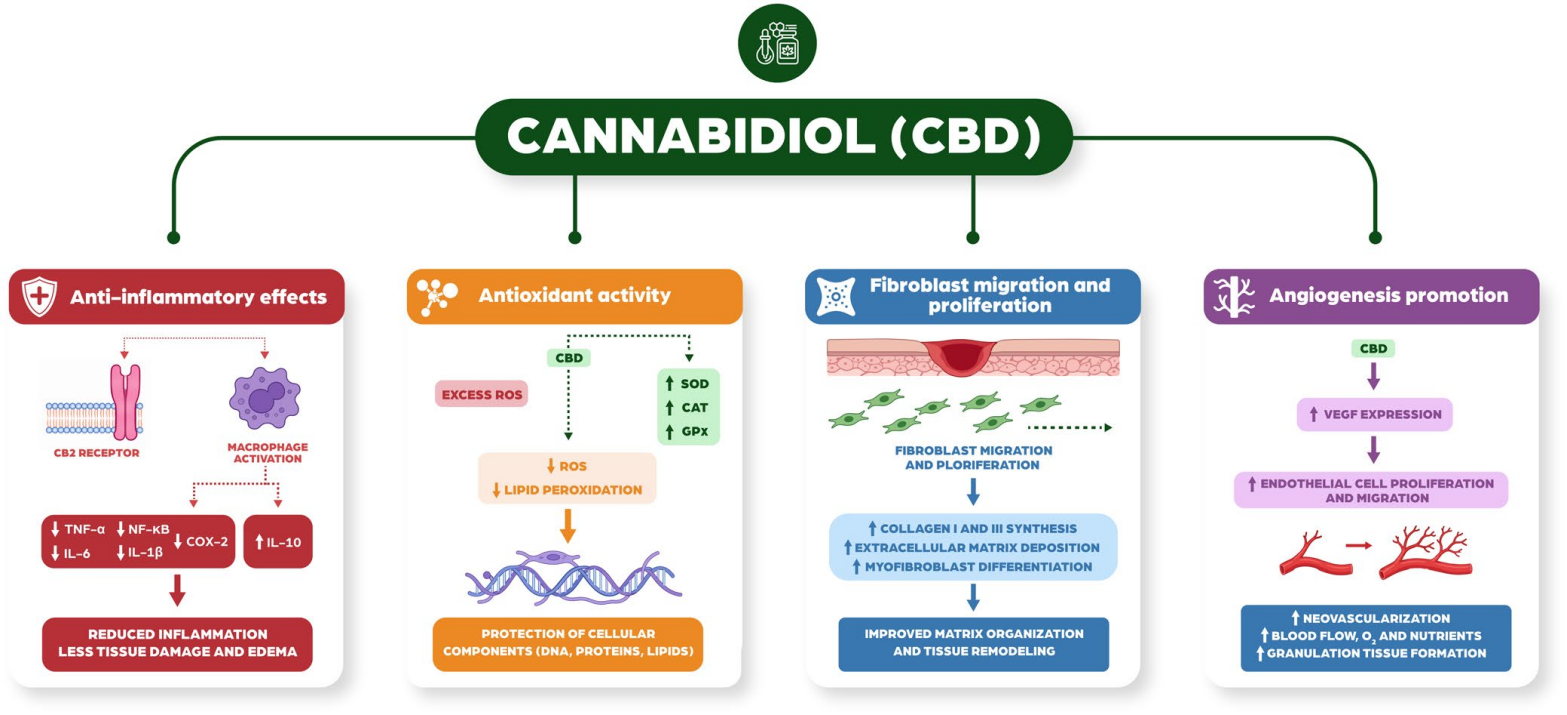
Schematic overview of the proposed mechanisms of cannabidiol (CBD) in cutaneous wound healing. CBD, cannabidiol; ROS, reactive oxygen species; SOD, superoxide dismutase; CAT, catalase; GPX, glutathione peroxidase; VEGF, vascular endothelial growth factor; TNF‐α, tumor necrosis factor alpha; IL, interleukin; NF‐κB, nuclear factor kappa B; COX‐2, cyclooxygenase‐2.

Nevertheless, the current evidence should be interpreted with caution. Future research should prioritize standardized dose–response studies, direct comparisons between CBD‐based therapies and current standards of care, optimization of delivery systems, and well‐designed randomized controlled trials involving patients with chronic wounds to better define the therapeutic role of CBD in clinical wound management.

## 5 Limitations

This study has several limitations that should be considered when interpreting the findings. It is important to clarify that, in the broader literature on cannabidiol and cutaneous wound healing, there is a predominance of review articles and a high proportion of in vitro investigations, reflecting the early and still developing nature of this field. However, within the scope of this systematic review, the available evidence directly addressing clinical wound healing outcomes or evaluating CBD as the primary intervention—either alone or in combination with delivery systems—remains limited. Furthermore, substantial methodological variability across the included studies may limit the comparability of results.

Therefore, there is a clear need for more robust and standardized experimental studies and, importantly, well‐designed clinical trials to better elucidate the efficacy and mechanisms of action of cannabidiol in the context of wound healing.

## 6 Conclusion

The body of evidence reviewed indicates that cannabidiol shows potential as a therapeutic agent in the context of wound healing, especially when combined with delivery systems that optimize its bioavailability and stability. Its effects appear to be related to the modulation of the tissue microenvironment, promoting conditions conducive to repair, although the magnitude of these benefits varies depending on the experimental model and the formulation used. In this regard, the use of pharmaco‐technical approaches, such as hydrogels and nanostructured systems, emerges as a promising strategy to enhance its efficacy. However, the consolidation of CBD use in clinical practice still depends on more robust and standardized investigations capable of clarifying its effectiveness in different biological contexts and establishing safe parameters for application.

## Author Contributions

João Henrique Ferreira França and Jaiurte Gomes Martins da Silva conceived and designed the study. João Henrique Ferreira França, Renale Beserra de Melo Freitas, Mariana Pereira Carvalho, and Geovanne Lucas Rodrigues Rosendo performed the literature search, study selection, and data extraction. João Henrique Ferreira França, Jaiurte Gomes Martins da Silva, Glicia Maria de Oliveira, Júlio César Claudino dos Santos, and Fernanda das Chagas Angelo Mendes Tenorio analyzed and interpreted the data. All authors contributed to the writing and critical revision of the manuscript and approved the final version.

## Funding

This study was financed in part by the Coordenação de Aperfeiçoamento de Pessoal de Nível Superior ‐ Brasil (CAPES) ‐ Finance Code 001.

## Conflicts of Interest

The authors declare no conflicts of interest.

## Data Availability

The authors have nothing to report.
